# A 5A's communication intervention to promote physical activity in underserved populations

**DOI:** 10.1186/1472-6963-12-374

**Published:** 2012-10-30

**Authors:** Jennifer K Carroll, Kevin Fiscella, Ronald M Epstein, Mechelle R Sanders, Geoffrey C Williams

**Affiliations:** 1 Department of Family Medicine, 1Family Medicine Research Programs, University of Rochester Medical Center, 1381 South Ave, Rochester, NY, 14620, USA; 2 Healthy Living Center, Center for Community Health, University of Rochester Medical Center, 46 Prince St, Rochester, NY, 14607, USA

**Keywords:** Patient centered communication, Self-determination theory, Physical activity, Primary care intervention, Underserved populations

## Abstract

**Background:**

The present study protocol describes the trial design of a clinician training intervention to improve physical activity counseling in underserved primary care settings using the 5As. The 5As (Ask, Advise, Agree, Assist, Arrange) are a clinical tool recommended for health behavior counseling in primary care.

**Methods/Design:**

The study is a two-arm randomized pilot pragmatic trial to examine a primary care clinician communication intervention on use of the 5As in discussion of physical activity in audio-recorded office visits in an ethnically diverse, low-income patient population. The study setting consists of two federally qualified community health centers in Rochester, NY. Eligible clinicians (n=15) are recruited and randomized into two groups. Group 1 clinicians participate in the training intervention first; Group 2 clinicians receive the intervention six months later. The intervention and its outcomes are informed by self-determination theory and principles of patient-centered communication. Assessment of outcomes is blinded. The primary outcome will be the frequency and quality of 5As discussions as judged by evaluating 375 audio-recorded patient visits distributed over baseline and in the post-intervention period (immediately post and at six months). Secondary outcomes will be changes in patients’ perceived competence to increase physical activity (Aim 2) and patients and clinicians beliefs regarding whether pertinent barriers to promoting exercise have been reduced. (Aim 3). Exploratory outcomes (Aim 4) are potential mediators of the intervention’s effect and whether the intervention affects actual enrollment in the community program recommended for exercise. The analysis will use repeated measures (in the form of recorded office visits) from each clinician at each time point and aggregate measures of Groups 1 and 2 over time.

**Discussion:**

Results will help elucidate the role of 5As communication training for clinicians on counseling for physical activity counseling in primary care. Results will explore the effectiveness of the 5As model linked to community resources for physical activity promotion for underserved groups.

## Background

With rapid increases in overweight, obesity, and related chronic conditions in the US and globally, [1,2] primary care faces an ongoing challenge and opportunity to translate promising physical activity interventions into practice [3]. Current US recommendations for healthy adults aged 18–65 entail either moderate-intensity aerobic physical activity for a minimum of 30 minutes/day for five days per week, vigorous intensity activity for a minimum of 20 minutes/day for three or more days per week, or a combination [4]. The World Health organization recommends that primary care be a cornerstone for physical activity promotion [5]. Primary care represents an important avenue for physical activity promotion in the US because about 11% of the population visits primary care physicians every month, 80% of adults visit a physician within a one-year period, [6] and more than 40% of adults over age 40 have had the same doctor for over 5 years [7].

Effective physical activity counseling in primary care is hampered by limited time and competing demands [8,9]. Primary care physicians spend a mean of 47 seconds in health maintenance and chronic care visits– though the time varies widely- providing combined diet and exercise advice to patients [10]. Not surprisingly, less than half of patients receiving such advice were able to recall it. Yet, spending even an extra minute in the visit discussing exercise can more than double patient recall [11].

Limited evidence suggests brief clinician counseling improves short- and long-term physical activity outcomes [12,13]. Specifically, clinician counseling as brief as one or two 3 to 5 minute sessions result in a significant increase in patients’ physical activity levels at eight months [13]. The 5A guidelines, in which clinicians Ask about (or Assess), Advise about, Agree upon, Assist and Arrange follow-up regarding patients’ behavior change efforts[14,15]—is a framework for brief counseling that may promote physical activity. Additionally, patient centered communication (PCC) -- in which clinicians elicit patients’ social contexts, values, expectations, and beliefs relevant to the target behavior, and support patient choice in whether, when and how to engage in physical activity—may also promote behavior change by patient participation, satisfaction, trust, and adherence to treatment plans [16-18]. Understanding each patient’s unique perspective, context and wishes is particularly salient for underserved populations whose socio-cultural contexts may differ substantially from those of their clinicians [19]. In the absence of patient input, clinicians may not fully appreciate the facilitators and barriers to physical activity that patients from diverse background confront.

Despite the potential impact, relatively little research has investigated how to promote effective physical activity counseling in primary care using the 5As or PCC, particularly for underserved patients. For underserved populations, counseling linked to accessible community-based resources may be a promising strategy.

### Conceptual framework

A challenge in applying health behavior research to clinical care is the existence of multiple overlapping theories of communication and behavior change. This project contains three common elements – motivation, skills, and support- in its design and measurement. Patients undertake behavior change when they experience internal motivation rather than external control, social support to change behavior, and the perceived competence with instrumental skills to accomplish the change and address barriers.

One theory that unifies these three elements is self-determination theory [20]. SDT is a general theory of human motivation, with the foundation that humans are innately motivated for personal growth and health. According to SDT, individuals have needs for autonomy, competence (e.g., feeling able to achieve a desired outcome), and relatedness to others [17]. Autonomy is defined as the need to have choice and volition in one’s behavior. Competence

Is defined as the need to feel optimally challenged and capable of achieving outcomes. Autonomy support has been linked to stronger intentions to be physically active, Rouse [21] long-term retention, higher levels of perceived autonomy for physical activity, initiation and maintenance of physical activity, greater weight loss in weight loss programs, [17,22] and perceived competence for physical activity [23]. A recent meta-analysis of 30 studies found a significant positive correlation between autonomy support and physical activity, with positive effect sizes in the small to moderate range for physical (0.08 to 0.39) and mental (0.22 to 0.37) health [24].

Additional information about self-determination theory and its application to the design of the intervention and choice of measures is in (sections Description of intervention and Evaluation and measures).

While self-determination theory is the over-arching theory used in this project, there are two other components incorporated into the conceptual framework. First, the 5A guidelines (“5As”) [14] focus on clinician completion of five specific tasks necessary to effectively counsel patients about health habits. Originally developed by the National Cancer Institute as the “4As” for smoking cessation, [14] the 5As have been endorsed by the US Preventive Services Task Force, [15] the Canadian Task Force on Preventive Care, [25] and national guidelines in the UK [26] and Sweden [27] as a unifying framework for behavioral counseling in primary care for non-tobacco health behaviors [7,15,28-30]. The 5As are a framework for clinicians to ASK about current behavior, ADVISE a change, ASSESS willingness to change and willingness to enroll in a community program supporting physical activity, ASSIST with goal-setting and ARRANGE follow-up. ASK is important for behavior change to explicitly identify physical activity as potentially in need of change; one’s physical activity level is usually unknown without specifically asking and rarely the main reason for seeking clinical care. ADVISE [15] is important for health behavior change by specifically linking physical activity recommendations to a person’s own health concerns or life context, in order to maximize motivation for change [31]. ASSESS promotes behavior change by the patient and clinician coming to common ground and collaborating on physical activity goals and strategies. ASSIST [15] is relevant for behavior change by offering additional resources, referral options, or practical problem-solving strategies help the patient secure the necessary support for physical activity change [15,32]. Finally, ARRANGE is important for behavior change by providing the opportunity to follow-up and re-evaluate one’s behavior change efforts and perhaps adjust the change plan [15].

The 5As have been endorsed as a unifying framework for behavioral counseling in primary care [7,15,28-30]. The 5As have been shown to increase healthy behaviors, positively influence mediators of behavioral change, and increase clinician communication skills about health behavior change [13,15,28-30,33-35]. The 5As are incorporated into the clinician training intervention’s design and measurement.

The second component in the conceptual framework is patient-centered communication (PCC) [36,37]. Patient-centered communication directly addresses barriers in counseling for underserved populations that may not be adequately captured in the 5As alone. Understanding patients’ social context might help narrow the gap in the social distance between patient and physician. For example, miscommunication can occur if clinicians give advice without understanding the patient’s life situation, without encouraging the patient to ask questions and take an active role, and without reinforcing the patient’s learning during the office visit by summarizing, checking, and verifying mutual understanding [38]. PCC improves trust, [16] motivation, adherence and control of some chronic illness [17,18] however its application to physical activity counseling is less well understood. PCC consists of several constructs; [39-44] for this project, we focus on the constructs overlapping with SDT of (1) autonomy support, defined as activating and involving patients in choices about their care [45] such that they feel supported and empowered, [46] and (2) understanding patients’ social context.

Table 1 summarizes how self-determination theory, the 5As, and patient-centered communication inter-relate in the project. In the intervention’s design, interactive group and peer discussions are incorporated into the training techniques to increase both clinician and patient motivation for physical activity discussion. This training technique relates to the 5As and PCC by increasing clinician motivation to ASK, ADVISE, and ASSESS their patient’s physical activity via the use of patient-centered communication skills of supportive listening and open-ended questions. The intervention is designed to provide a choice of options and community resources for clinician to ASSIST and ARRANGE referral for physical activity, thus aiming to increase clinician autonomy supportiveness and competence to counsel. In the assessment and measurement, SDT, the 5As and PCC inter-relate in the analysis of the discussions via ratings of the content of the physical activity discussions (using the 5As), and the quality of the physical activity discussions (whether they were autonomy supportive-SDT, and whether they explored the clinician made supportive statements, and verified understanding and agreement-PCC).

**Table 1:** Summary of intervention components and corresponding conceptual framework

	**Self-determination theory**	**5As**	**Patient-centered communication (PCC)**
Design of intervention (key concepts)	· Promoting autonomy supportive skills for clinicians when counseling patients about physical activity	· Use of 5As for physical activity counseling	· Understanding patients’ social context
			· Offering support
			· Encouraging patient participation
	· Increasing clinician perceived competence to counsel		
Intervention training (curriculum components)	· Interactive discussion on strategies to increase both patient motivation for physical activity and clinician motivation to raise the topic	· Introduction, repetition, and reinforcement of each of the 5As via didactic presentation, role play, and standardized patient feedback	· Role play and group discussion to develop and reinforce supportive listening and open-ended questions about physical activity
	· Offering a choice of community resources for referral		
	· Offering a choice of optional electronic health records tools and eliciting ongoing feedback		
			· Use of standardized patients to give feedback to clinicians on PCC skills
Assessment/measurement (clinician perspective)	Clinician surveys and interviews	Clinician interviews asking about recall of 5As	Clinician surveys and interviews
Assessment/measurement (patient perspective)	Patient ratings of autonomy support of clinicians, perceived competence for physical activity	Patient report of 5As discussions of physical activity	Patient ratings of trust and satisfaction with their relationship with their clinician; patient interviews on communication skills of their clinicians
Assessment/measurement (blinded coder)	Coding of autonomy supportiveness (global rating and for each A)	Coding of content and quality ratings for the 5As	Coding of supportive statements, exploration of patient’s social context related to physical activity, encouraging questions, verifying understanding and agreement

The purpose of this paper is to describe a study protocol of a clinician training intervention to improve physical activity counseling in underserved primary care settings using the 5As. Aim 1 of the project assesses whether a 5As physical activity communication training intervention increases communication skills during visits underserved patients in the post-intervention period (immediately post and at one year compared to baseline). Aim 2 assesses whether the 5As communication training intervention improves patients’ perceived competence for physical activity. Aim 3 assesses whether the 5As communication training intervention improves clinicians’ autonomy support for physical activity with their patients. Aim 4 assesses whether clinicians and patients perceive the 5As counseling to be feasible and sustainable and whether the communication training addresses pertinent barriers to promoting physical activity.

The hypotheses are as follows:

H1: Clinicians who complete the communication intervention will increase their use of all 5As for visits in which the topic of physical activity is raised as judged by comparing the audio-recordings of office visits in the post-intervention period (immediately post and at one year) compared to baseline.

H2: The intervention will increase clinician autonomy supportiveness when counseling about physical activity.

H3: The intervention will increase patient perceived competence for adopting physical activity.

H4: Specific patient recall of the Assist and Arrange will be associated with the greatest patient perceived competence to adopt physical activity compared to recall of Ask, Advise, or Assess.

### Conceptual framework

A challenge in applying health behavior research to clinical care is the existence of multiple overlapping theories of communication and behavior change. This project contains three common elements – motivation, skills, and support- in its design and measurement. Patients undertake behavior change when they experience internal motivation rather than external control, social support to change behavior, and the perceived competence with instrumental skills to accomplish the change and address barriers. One theory that unifies these three elements is self-determination theory [20]. We use self-determination theory in the design of the intervention and choice of measures (described further in sections 2.11 and 2.14).

While self-determination theory is the over-arching theory used in this project, there are two other components incorporated into the conceptual framework. First, the 5A guidelines (“5As”) [14] focus on clinician completion of five specific tasks necessary to effectively counsel patients about health habits. Originally developed by the National Cancer Institute as the “4As” for smoking cessation, [14] the 5As have been endorsed by the US Preventive Services Task Force, [15] the Canadian Task Force on Preventive Care, [25] and national guidelines in the UK [26] and Sweden [27] as a unifying framework for behavioral counseling in primary care for non-tobacco health behaviors [7,15,28-30]. The 5As are a framework for clinicians to ASK about current behavior, ADVISE a change, ASSESS willingness to change and willingness to enroll in a community program supporting physical activity, ASSIST with goal-setting and ARRANGE follow-up. The 5As have been endorsed as a unifying framework for behavioral counseling in primary care.[7,15,28-30] The 5As have been shown to increase healthy behaviors, positively influence mediators of behavioral change, and increase clinician communication skills about health behavior change [13,15,28-30,33-35]. The 5As are incorporated into the clinician training intervention’s design and measurement.

The second component in the conceptual framework is patient-centered communication (PCC) [36,37]. Patient-centered communication directly addresses barriers in counseling for underserved populations that may not be adequately captured in the 5As alone. Understanding patients’ social context might help narrow the gap in the social distance between patient and physician. For example, miscommunication can occur if clinicians give advice without understanding the patient’s life situation, without encouraging the patient to ask questions and take an active role, and without reinforcing the patient’s learning during the office visit by summarizing, checking, and verifying mutual understanding [38]. PCC improves trust,[16] motivation, adherence and control of some chronic illness [17,18] however its application to physical activity counseling is less well understood. PCC consists of several constructs; [39-44] for this project, we focus on the constructs overlapping with SDT of (1) autonomy support, defined as activating and involving patients in choices about their care [45] such that they feel supported and empowered, [46] and (2) understanding patients’ social context.

Table 1 summarizes the application of self-determination theory, the 5As, and patient-centered communication in the project.

## Methods

### Study design

**Figure 1:**
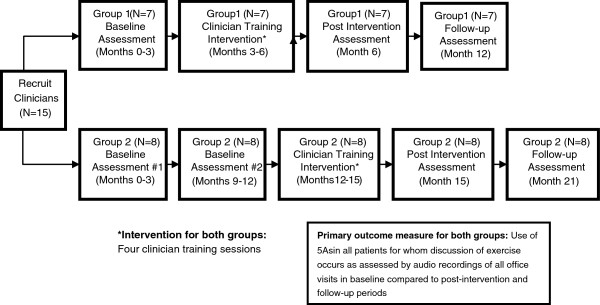
Study Schema.

The study methods described herein have been developed and informed by the CONSORT criteria and by other work in the field [12,13,47-49] Figure 1. 

The project is a two-arm randomized pragmatic trial to pilot a communication training intervention for primary care clinicians (n=13) on use of the 5As to promote physical activity in underserved patients. The intention of training focuses on clinicians to communicate effectively using the 5As and patient-centered communication skills firmly linked to a theory of human motivation (Aim 1). Clinicians are randomly assigned to two groups. Group 1 participates in the training intervention first; Group 2 receives the intervention six months later. The primary outcome is the frequency and quality of clinician use of the 5As during routine office visits, post-intervention compared to baseline. Secondary outcomes and potential mediators are changes in patients’ perceived competence to adopt physical activity (Aim 2) and clinician autonomy support of physical activity with their patients (Aim 3). Given the myriad of barriers reported in the literature, another secondary outcome is whether clinicians believe that the intervention addresses pertinent barriers to promoting exercise (Aim 4). Exploratory outcomes are other potential mediators of the intervention’s effect and the effect of the intervention on actual physical activity levels in a subset of participants. The schema shows the overall study design and timeline.

### Study Setting

The study population consists of patients and clinicians at two federally qualified community health centers in inner-city Rochester, NY. The health centers provide a full range of primary care services to a predominantly low-income and/or uninsured, ethnically and culturally diverse patient population including African, African-American, Asian, Asian-American, Eastern European, Hispanic, and several other foreign-born immigrant and refugee patients. There are 15 family physicians, two nurse practitioners, and two physician assistants at the two health centers. In the US, both nurse practitioners and physician assistants have graduate-level education and are licensed and certified to treat a variety of physical and mental health conditions and provide health behavior counseling.

### Participant eligibility

Patient and clinician eligibility criteria are shown in Table 2. Patient eligibility is determined by the Research Assistant reviewing clinicians’ schedules with a checklist of inclusion and exclusion criteria. If eligibility is uncertain, the Research Assistant checks with office staff. Patients are excluded if they are deemed inappropriate for participation due to an acute or unstable medical condition or if they are unable to provide informed consent due to a language barrier, limited literacy, and/or cognitive concerns.

**Table 2:** Inclusion and exclusion criteria

	**Inclusion criteria**	**Exclusion criteria**
Patients	**·** Currently enrolled patients at Westside Health Services, Inc.)	**·** Have a life-threatening or acute medical problem which precludes participation
	**·** Scheduled for a routine, follow-up, or health maintenance office visit	**·** Unable to read and understand English
	**·** Scheduled to see a participating clinician	
	**·** 18 years of age or older	
	**·** Able to provide written informed consent	
	**·** Have one or more stable medical conditions for which activity is not contraindicated (e.g., asthma, patients undergoing chemotherapy, diabetes, hypertension, stable cardiovascular, neurological, or psychiatric disease)	
Clinicians	**·** Practicing clinicians (physicians, physician assistants, or nurse practitioners) at community health center organization	**·** Planning to move or relocate to another practice in the study period
		**·** Serving as study investigators or advisors

### Recruitment of clinician participants

The principal investigator presents an overview of the study’s objectives, design, and time frame to eligible clinicians at a weekly clinician meeting. Each clinician’s full participation requires the clinician to 1) complete a 10 minute written baseline assessment, 2) attend four one-hour training sessions held during regularly scheduled provider meeting times over a four-month period, 3) consent to having 10 patient visits audio-recorded at baseline and 15 at post-intervention, and 4) complete a post-intervention survey and a 20 minute individual interview. Based on estimates from a previous pilot project, we anticipate a 10 patient visits at baseline should be sufficient; however, if the visits do not contain any discussions of physical activity, clinicians are told that the baseline assessment may consist of > 10 visits. In addition to clinicians’ informed consent, research staff explain that patient informed consent is obtained prior to any recording. For patients providing informed consent, an audio-recorder is placed unobtrusively in the exam room. Each clinician receives $60 per completed hour of the four hours of training and $5 per recorded visit. The principal investigator also explains the overall goals and procedures of the study to all clinical staff at their regularly scheduled team meetings. Staff are encouraged to forward any inquiries from patients about the study to members of the study team. The clinical practice receives a $2000 stipend for use of the facility and staff time. Clinician participants then provide written informed consent.

### Procedure for randomization

A concealed randomization procedure stratified by health center location and clinician type (physician, nurse practitioner, or physician assistant) is used to balance randomization between both groups. A series of treatment assignments in a block of five is generated by the study statistician in advance of the intervention and placed in a file accessible to the Research Assistant responsible for administering the group assignment to each clinician. Clinicians are notified of their randomization assignment by the Research Assistant immediately after providing informed consent by opening a sealed envelope with the assigned intervention category for each individual: Group 1 (Early) or Group 2 (Wait-list). Neither study clinicians nor research staff are blind to the assignment of clinicians to the early or wait-list training groups.

### Clinician baseline assessment

Prior to the training intervention, clinician participants from both groups complete a baseline assessment consisting of (1) a brief survey to obtain baseline demographic information, experience and confidence with physical activity counseling, attitudes and beliefs about incorporating it into their practice, and knowledge of community resources for physical activity for their patients, and (2) a baseline assessment of 10 audio-recorded office visits.

### Patient participant recruitment and enrollment

When potentially eligible patients enter exam rooms for their visit, a nursing assistant mentions the project to see if they might be interested in participating. The Research Assistant then obtains written consent from eligible and interested patients. As part of the informed consent process, patients are told that they are not obliged to deliberately discuss physical activity. The visit is then audio-recorded. As with the clinician informed consent procedure, patients are explicitly informed that if they agree to participate, the recorder is placed unobtrusively in the exam room. The consent process takes about five minutes and is designed for minimal disruption of office flow and schedules. Patients are recruited until there is adequate baseline information about physical activity discussions.

### Patient post-visit survey

After the recorded visit, a brief face-to-face survey is administered to the patient participants. Items include socio-demographics data (age, gender, marital status, race and ethnicity, highest educational level attained, and insurance status), the SF-12, and other measures described in (sections Evaluation and measures and Exploratory outcome measures). The survey takes about 15 minutes. Patients then complete a 5–10 minute interview asking about their perspective on the content, adequacy, and clarity of communication about physical activity counseling.

Patients are compensated a total of $20 ($10 for participating in the audio recording portion and $10 for their time completing the survey and interview).

After the baseline assessment is completed for Group 1 clinicians and their corresponding patients, Group 1’s training intervention begins.

### Description of intervention

The intervention consists of four clinician training sessions, described below. Sessions 1–3 are conducted as a group; Session 4 is individually-based. Each session is facilitated by the principal investigator.

#### Curriculum for clinician training intervention

Clinicians are trained using didactic materials, skills/competency checklists, sample “scripts”, role play and cognitive rehearsal -- procedures regarded as effective techniques for communication training [37,50,51]. Standardized patients (actors) are used to portray realistic clinical situations, offer critique and feedback to each clinician, and assure that clinicians achieved the requisite skills.

#### Clinician training session 1

The objectives of Session 1 are to discuss 1) current evidence-based recommendations for physical activity, 2) contraindications to physical activity, 3) overview of the 5As, and 4) patient-centered communication and its use in physical activity counseling. Session 1 consists of a didactic presentation and an interactive group discussion. Consistent with self-determination theory and patient-centered communication, the discussion seeks to enhance clinicians’ understanding of their patients’ personal and social contexts for physical activity via counseling skills to elicit patient motivation for physical activity. The rationale for this is that understanding thepatients personal and social contexts for PA is consistent with supporting patient autonomy (by eliciting and acknowledging their feelings and perspectives about how their contexts might support or impede change) and competence (e.g. whether one’s own context influences their feelings of effectiveness in achieving their exercise goals.) The discussion also aims to enhance clinician motivation and competence to counsel by encouraging the group to discuss realistic ways to discuss physical activity despite the myriad challenges, barriers, and competing demands. All physical activity recommendations in the study are based on current physical activity national guidelines at the time of the intervention [52]. “To accumulate 30 minutes of moderate intensity physical activity over the course of most, preferably all, days of the week.”

#### Clinician training session 2

Session 2 consists of a brief review of the 5As, followed by an introduction to electronic tools developed to supplement the 5As and patient-centered communication for physical activity counseling using the practice electronic medical record. The tools are: 1) a History of Present Illness template, in which clinicians can view prompts to physical activity questions and answers, barriers, goals for change, and use pre-developed text to enter directly into the progress note , 2) a Social History box for entering physical activity data on type, duration, frequency, and intensity, 3) a Preventive Medicine page in the patient’s chart containing a generic activity prescription available for editing and customizing, 4) an Order Sets page in the chart, activated through one or two screen clicks for retrieving patient education handouts, and 5) a Referrals link in the chart, activated by one click, to make referrals to the community healthy living program. Clinicians receive a resource list with information on free or low cost community programs to consider as referral options for physical activity. Clinicians are taught how to use the Referrals Tab to refer patients to a community program for lifestyle change and physical activity that is available on-site at the health center. In order to support clinician competence and motivation to discuss physical activity, a variety of tools are provided to encourage flexibility in documentation of physical activity in the electronic health record and to accommodate different work styles. The local resource list is developed and provided to increase clinician confidence to counsel through awareness of available community options for physical activity. Additional file 1 shows screen shots of the five tools.

#### Clinician training session 3

The objective of session 3 is to implement the 5As including referral to an appropriate community resource if the patient was willing to go, while working with a standardized patient and observed by a peer. Session 3 also emphasizes the theoretical concepts of eliciting patients’ social context and being autonomy supportive by eliciting patients’ perspectives, preferences, and willingness to change. Clinicians are divided into pairs with each person taking turns conducting a visit with a standardized patient, debriefing with the standardized patient and the peer, and then repeating the cycle with a different standardized patient. Each standardized patient is trained according to case vignettes which have been developed and pre-tested with the principal investigator to portray common patient presentations and clinical scenarios for which physical activity discussion would be appropriate.

#### Clinician training session 4

The goal of session 4 is to reinforce and rehearse all of the aforementioned elements of the training and receive individual intensive feedback with a standardized patient. A standardized patient meets with each clinician individually to perform an assessment of skills acquired from the training according to a predefined checklist of core competencies. The standardized patient for session 4 is designed to portray a challenging patient with multiple medical and psychosocial conditions and barriers to exercise. The feedback emphasizes the importance of supportive listening to understand patients’ life contexts and challenges to activity, providing realistic problem-solving techniques to patients, and explicit mention of available community resources for physical activity. The standardized patient gives each clinician feedback on their 5As and PCC skills using a standardized checklist of competencies and open-ended, qualitative feedback.

### Post-intervention clinician assessment

The post-intervention clinician assessment occurs immediately upon completion of the intervention (or at approximately 6 months from the baseline assessment) and 6 months follow-up (or 12 months from the baseline) in the same manner described previously. Clinicians complete post-intervention and follow-up assessments of 15 audio-recorded office visits, divided across the two time points in the same manner as described in section 2.8. Group 1 clinicians complete a survey asking about the feasibility of the intervention, and individual brief interviews are conducted.

### Group 2 (wait-list)

The clinician baseline assessment for Group 2 is identical to that for Group 1. The Group 2 assessment occurs at 0–3 months (concomitant with the Group 1 clinician baseline) and again at 9–12 months (concomitant with the Group 1 post-intervention assessment.)

Group 2’s clinician training intervention occurs in the same manner as described in the section, Description of Intervention (section 2.11) for Group 1. Standardization of both Group 1 and Group 2’s training intervention sessions is assessed by audio-recording all sessions and ensuring that the key objectives of each session are met using a pre-developed checklist and via rating by a trained research staff member not involved in delivering the intervention.

Group 2’s Post-Intervention Assessment occurs immediately post-intervention and at six months post-intervention in the same manner as described previously for Group 1 (section Post-Intervention Clinician Assessment). As with Group 1, Group 2 clinicians also complete a survey asking about the feasibility of the intervention and individual exit interviews.

### Evaluation and measures

#### Primary outcome measure: 5As score

The primary outcome measure is the 5As score, a score of the frequency and quality of 5As discussions about physical activity. Based on our previous pilot study and others’ published work [11,15,53,54] we developed a coding form to capture the 5As from audio-recorded office visit discussions. The 5As score has two components: 1) the sum of each A of the 5As occurring in each office visit when the topic of physical activity is raised (range 0/no As used to 5/all As used) and 2) a rating of the quality of each A when it occurs (ranging from 1–3 based level of detail from minimal to intensive). The 5As score is measured at baseline, immediately post-intervention, and at six months follow-up for Groups 1 and 2. The 5As score is assessed by trained research staff blinded to clinician and time point.

#### Secondary outcome measures

The Perceived Competence Scale (PCS) measures the self-determination construct of patient perceived competence in their ability to increase their level of physical activity [17]. The PCS is a four item (Alpha reliability = 0.90) validated psychometric instrument to measure a person’s feelings of competence at carrying out a physical activity [55,56]. The PCS is administered to patients at baseline, post-intervention and follow-up as part of the post-visit survey’.

The Modified Health Care Climate Questionnaire (mHCCQ) [17] is a patient reported measure of the degree to which the clinician provides autonomy support, a construct derived from self-determination theory shown to be modifiable in health behavior interventions. The mHCCQ is a six item validated psychometric instrument that has been shown to be associated with behavior change for smoking cessation, weight loss and maintenance, and exercise. The mHCCQ measures patient perceptions of providers as autonomy supportive versus controlling and has been validated for use in primary-care offices (Alpha reliability is 0.92) [57]. The mHCCQ is administered at baseline, post-intervention and follow-up.

Clinicians’ perspectives on the intervention’s feasibility, sustainability, and learning objectives acquired, is assessed three ways: (1) via a survey administered at baseline and post-intervention, (2) via a process evaluation throughout the training period, and (3) via individual clinician post-intervention interviews. For the survey, we use a previously published survey from the Physical Activity for Life (PAL) study, which is a 20 item face-valid questionnaire to explore clinician impressions of the effectiveness of the intervention [13]. The survey contains items asking about clinician perspectives on feasibility, knowledge, and skills gained from the intervention. Ongoing process evaluation data are collected throughout the intervention’s training sessions in the form of field notes and audiorecorded feedback from clinicians during the training sessions. At the conclusion of the study, the principal investigator and research assistant conduct individual interviews to elicit clinician perspectives on feasibility of the intervention, relevance of training objectives, and suggestions for improvement.

#### Exploratory outcome measures

Moderation variables consist of patient factors and visit factors. Patient factors are socio-demographic variables, body mass index, baseline health, [58,59] health literacy[60], co-morbidities, and length of relationship with their clinician. Visit factors consist of type of visit, measured categorically using the clinic schedules (e.g. routine follow-up, health care maintenance) and competing demands measured by the 1) number of topics discussed and 1) number of health behaviors discussed.

Mediation variables consist of patient perceived competence for physical activity (using the PCS), clinician autonomy support (using the mHCCQ) for physical activity, and patient recall of 5A using the Physical Activity Exit Interview (PAEI) [47]. The PAEI is a 12-item survey administered to patients after their visit asking them to recall specific content (Yes/No) to questions corresponding to each A for physical activity, such as “Did your doctor advise you to become more physically active?”, “Did your doctor discuss difficult situations you might encounter or problems you might have in trying to become more physically active?”, and “Did your doctor state that he/she is planning to discuss your physical activity on a future visit?”

### Data management

All questionnaires and measurements will be collected and entered into a secure protected database by the Research Assistants. Data will be entered on scannable forms and electronically sent to an Access database. After scanning, data will be audited visually for errors. SAS statistical packages are used for the analyses. Unless otherwise stated, all statistical tests will be performed at the two-tailed 5% level of significance. Likewise, 95% confidence intervals will be constructed for estimation of effects (e.g., difference in mean 5A scores across time points).

#### Assumptions

The assumptions underlying all statistical analyses will be thoroughly checked using appropriate graphical and numerical methods [61,62]. If outliers or influential data are detected, the accuracy of the data will be investigated. If no errors are found, analyses may be repeated after removing these cases to evaluate their impact on the results. However, the final analyses will include these data points.

#### Missing values

In the event that missing data occur, we will attempt to contact participants and obtain the data or to find out why the questionnaires or items are missing, and document the reasons for missing data. The planned analyses employ a mixed models approach that do not require complete data on all participants but make the assumption that data are missing completely at random; this assumption will be examined.

### Statistical analyses

**Figure 2:**
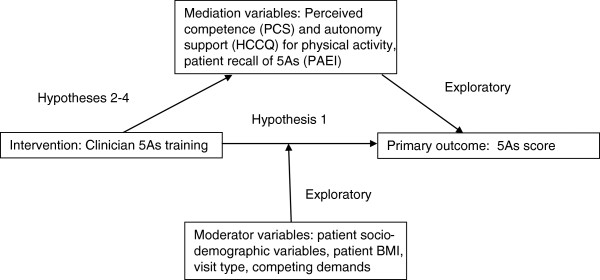
Mediators and moderators to be assessed in exploratory analyses.

The primary outcome measure will be the 5A score as described in (section Primary outcome measure: 5As score). The primary analysis will use a mixed effect model to compare mean 5As scores between and within each group for each of the three time points: baseline, immediately post-intervention, and follow-up. The primary hypothesis (H1) is: Clinicians who complete the communication intervention will increase their use of all 5As for all visits in which the topic of physical activity is raised as judged by audio-recordings of office visits in the post-intervention period (immediately post and at one year) compared to baseline. Figure 2 shows the analysis plan for hypothesis testing and exploratory outcomes.

The clinicians will be included as a random effect, and intervention group, time, and baseline 5A scores will be included as fixed effects. The interaction between treatment and time will be used to assess whether the treatment effect (of the intervention) changes with time.

Secondary analyses include similar modeling approaches for the secondary outcome variables: autonomy support (mHCCQ), patient perceived competence (PCS) and clinician feasibility (PAL). Clinician feasibility will also be qualitatively analyzed using the process evaluation data and individual clinician exit interviews. The secondary hypotheses (H2-H4) are as follows:

H 2: The intervention will increase clinician use of the PCC construct autonomy supportiveness as assessed by the mHCCQ in the post-intervention period (immediately post and at one year) compared to baseline.

H 3: The intervention will increase patient perceived competence (accessed via the PCS) for adopting physical activity.

H 4: Specific patient recall of the Assist and Arrange will be associated with the greatest patient perceived competence to adopt physical activity compared to recall of Ask, Advise, or Assess.

Hypotheses 2–4 will be tested by measuring the changes in the mHCCQ, PCS, and PAEI scores, respectively, for patients seen by clinicians in Group 1 in the post-intervention period (immediately post and at one year) compared to baseline. compared to the Group 2 baseline and Group 1 baseline. We will also compare change in the 5A scores for Group 2 post-intervention compared to Groups 1 and 2 baseline scores.

Exploratory analyses will use a similar approach to that used for the primary analysis, to examine whether the primary outcome (5A score) was mediated by patient perceived competence (PCS score), clinician autonomy supportiveness (mHCCQ score), or patient recall of the 5As (PAEI score). Additional exploratory analyses will examine the role of selected factors that may moderate the relationship between the intervention and the 5A score: continuity of care, patient socio-demographic variables, patient BMI, baseline health, health behaviors, health literacy, and competing demands. Regression-based statistical models will be constructed and examined. The Diagram below shows the inter-relationship of the mediating and moderating factors to be explored in this analysis.

### Sample size and power

The primary goal of this study is to evaluate the influence of a clinician communication intervention on use of the 5As in communication about physical activity in the post-intervention period (immediately post and at one year) compared to baseline as assessed by audio-recorded data. The sample includes a planned maximum of 15 clinicians (eight in Group 1 and seven in Group 2) measured at three (Group 1) or four (Group 2) time points. The measurements consist of a total of 25 audio-recorded patient visits from each clinician distributed across each of the evaluation points: approximately 10 recordings at baseline and seven or eight each at post- and follow-up points. Of the planned 375 recorded visits, we estimate up to 20% may be excluded from the analysis due to the patient or clinician shutting off the recorder, or equipment malfunction, thus leaving approximately 300 recorded visits (about seven per clinician per time point, or 20 total per clinician) for analysis. Pilot data with 46 recorded visits from eleven physicians showed that the average 5A score was 0.74, with a standard deviation of 1.06. Thus, with 15 clinicians and 20 patients per clinician, we will have 80% power to detect a difference of 0.54 between Groups 1 and 2, using a two-sided t-test at 0.05 significance level.

The final analyses will use a mixed effects model to incorporate correlations between the observations made by the same physician. The variance in 5A scores taken from patients within a single physician's patient panel will likely be smaller than the variance of scores taken from patients between different physicians, i.e. the intraclass correlation coefficient (the ratio of between-physician variance to the sum of between- and within-physician variances) will be larger than zero. We have conducted a series of power analyses taking this clustering into effect using a Mann–Whitney two-sample t-test of means.

### Process evaluation

Process evaluation data will be analyzed to examine the feasibility and acceptability of the intervention. Qualitative data consist of field notes of all clinician training sessions, observation notes on recruitment procedures and cooperation between clinical and research staff, correspondence notes and meeting minutes between community partners and study staff, notes from study staff and faculty research meetings, patient post-study interviews and focus groups, and transcribed clinician interviews. A multidisciplinary team will conduct the qualitative analysis using grounded theory, [63,64] a coding-editing approach, in two phases. In the first phase, members of the team (undergraduate students) will systematically code all qualitative data and meet regularly with the principal investigator, who has expertise in qualitative research, to review codes, identify and resolve discrepancies, and discuss emerging concepts. For the second phase, a multidisciplinary group of faculty and graduate-level trainees will review the codes and concepts to develop the key themes related to the intervention’s feasibility and acceptability. Quantitative process measures consist of clinician attendance at all training sessions, clinician fidelity to the intervention (measured by audio recordings), other clinic staff participation in patient recruitment, patient participation rate, reasons for patient refusal or ineligibility, patient completion of all study measures, patient follow-through at the community program referral and pre-post clinician ratings of usefulness or recommendation of the community resources to their patients.

### Assessing cross-contamination

We will use the audio-recorded data to measure cross-contamination in Group 2 clinicians to evaluate the extent to which they could be influenced by the intervention. Specifically, we will assess changes in Group 2’s 5A scores from two baseline measurements at months 1–3 and months 12–15. Trained research staff, blinded to the clinician’s group assignment, will listen to the baseline audio-recorded sessions of Group 2 clinicians and use pre-developed intervention adherence checklists to evaluate whether Group 2 clinicians incorporated specific intervention-related materials into their visits and whether they explicitly used the 5As or patient-centered communication constructs in their visits. Group 2 clinicians will also be asked directly in their post-study individual interviews whether they were aware of the study activities from Group 1 and the extent to which it influenced their counseling, if applicable.

### Data safety monitoring plan

The study protocol will be monitored by the Principal Investigator for safety via weekly contact with the research staff, with resolution of any data safety issues that arise. The protocol will be monitored by tracking the status of activities in the study’s phases of recruitment, enrollment, coding of visits, and data entry from surveys. All data materials will be kept confidential, stored and locked in the Principal Investigator’s private office, identifiable only by coded numbers.

### Design limitations and other considerations

Maximizing clinician adherence, consistency and fidelity to any intervention in a clinical setting is challenging. Steps to address these issues are to give positive feedback, regular updates, encouragement and incentives for the clinicians, nursing staff, and organization. It is possible that improvement after intervention will be minimal and difficult to sustain given the competing needs and challenges inherent in working with underserved patient populations. Thirdly, cross-contamination, spillover, and/or Hawthorne effects are concerns in this study; we will assess these issues in our analysis taking advantage of the two group design feature and directly audio-recorded, objective data source as well as post-study clinician interviews asking them to estimate the extent to which they may have been biased by the presence of the recorder. Finally, this study is unable to follow a cohort of patients pre- and post- intervention, though it is possible to look at aggregate baseline- and post-intervention patient-level factors that may affect use of the 5As. Given the relatively small scale of this project, this limitation is a recognized trade-off for practical and logistical considerations. The first priority for this project is to look at clinician implementation issues. Patient outcomes are secondary and exploratory in this study but will be key in future work.

## Discussion

Conservative estimates place direct health care costs of sedentary behavior at 24.3 billion dollars per year and of obesity at 70–147 (2008 est.) billion dollars per year [65,66]. Recent data suggest that behavioral risk factors such as inadequate physical activity account for a greater portion of health disparities than was previously thought [67]. Disadvantaged groups are less likely to have sufficient physical activity [52,68] and more likely to suffer related adverse health outcomes [69-71]. Disadvantaged groups have not been well represented in clinical trials to promote physical activity, though this is beginning to change [72-75]. Primary care has potential for successful interventions focused on disadvantaged groups because it offers a trusted source of continuity across an individual’s lifespan, often serving entire families or generations. Safety net clinics, particularly federally qualified health centers, hold promise for reducing disparities because they provide the largest proportion of primary health care services to medically underserved and vulnerable populations [76]. Federally qualified health centers serve 20 million patients—this is expected to double to 40 million patients under the Affordable Care Act [77]. Primary care clinicians are on the front lines of managing medical complications related to inadequate physical activity [78]. Therefore there is an urgent need to accelerate the translation of physical activity interventions into community-based and primary care settings, which this project will evaluate.

The 5A guidelines, in which clinicians Ask about (or Assess), Advise about, Agree upon, Assist with and Arrange follow-up regarding patients’ behavior change efforts [14,15] are recommended as an evidence-based clinical tool for health behavior counseling. The contribution of the present project is expected to be new knowledge of how a 5As intervention can be implemented to promote physical activity in disadvantaged patients. This contribution will be significant because it will develop and refine effective and efficient methods and strategies to implement evidence-based physical activity interventions into clinical practice settings. Knowledge gained from this project will directly inform key principles driving the Patient Centered Medical Home initiative, specifically by investing in training and redesigning the primary care workforce, intensifying health behavior change as a key feature of the clinical care model, and strengthening clinical-community partnerships.

The proposed research advances links between the 5As and self- determination theory (SDT) around common elements of patient autonomy [15,79] and change in physical activity. This project represents an interface between T3 (moving evidence-based guidelines into health practice through implementation research) and T4 (evaluation of health outcomes on real-world, population health practice) translational research and will inform this avenue of relatively under-developed research in the field to date [80-82]. We expect this strategy will have great potential for long-term adoption and sustainability.

## Conclusions

This project aims to improve clinician-patient communication about physical activity in underserved patients in primary care settings. It seeks to improve the frequency and quality of physical activity discussions by training clinicians to Ask about, Advise, Agree upon, Assist and Arrange a plan for physical activity. From a population perspective, increasing the prevalence of clinician communication about physical activity could greatly affect productivity, quality of life, mortality, and health costs in the United States. Results from this study will inform larger clinical trials that incorporate greater numbers of clinicians, patients, and practice sites to test the effects of combined patient-clinician communication and community-based exercise programs on physical activity and patient outcomes in underserved populations.

## Competing interests

The authors declare that they have no competing interests.

## Authors' contributions

JC conceived of the study, oversaw the design and implementation of study, and drafted the manuscript. KF, RE and GW participated in the design of the study and provided feedback and edits to drafts of the manuscript. MS participated in the implementation of the study and drafting and edits of the manuscript. All authors read and approved the final manuscript.

## Pre-publication history

The pre-publication history for this paper can be accessed here:

http://www.biomedcentral.com/1472-6963/12/374/prepub

## Supplementary Material

Additional file 1 Electronic Health Records Tools Screen Shots. Click here for file
