# Supplementary material for: A 5A's communication intervention to promote physical activity in underserved populations
Source: BMC Health Serv Res. 2012 Oct 30;12:374. doi: 10.1186/1472-6963-12-374 (PMC3506481; doi:10.1186/1472-6963-12-374)
Supplement: Additional file 1 — Electronic Health Records Tools Screen Shots. [file 1472-6963-12-374-S1.docx]

**Appendix A: Electronic Health Records Tools Screen Shots**

**Figure 1: History of Present Illness template for physical activity**

**
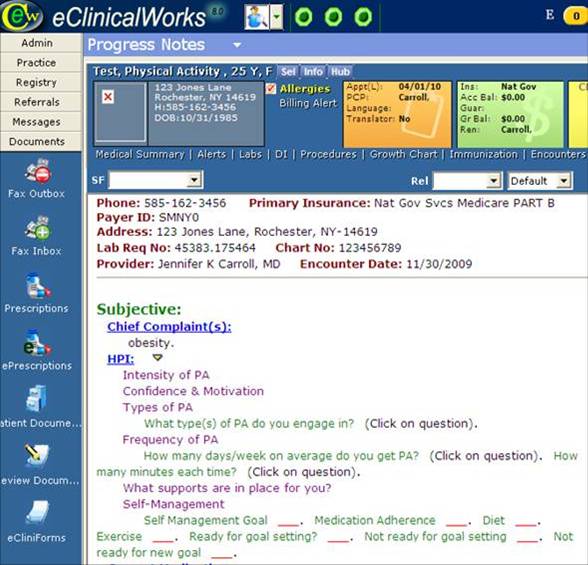
**

**Figure 2: Social History box**

**
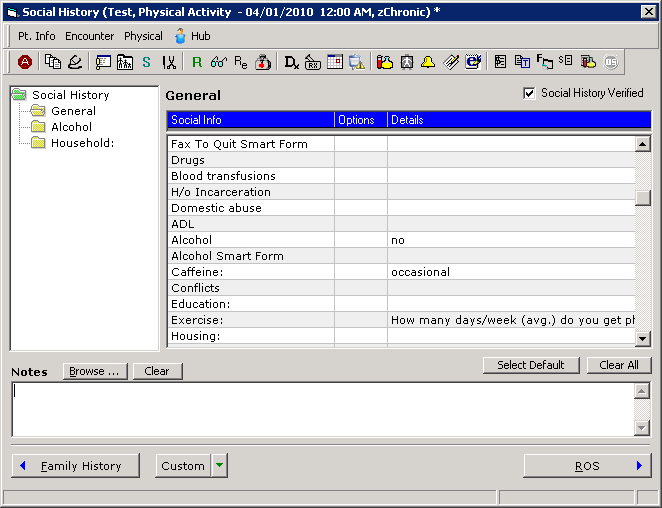
**

**Figure 3: Preventive Medicine Link for physical activity prescription**

**
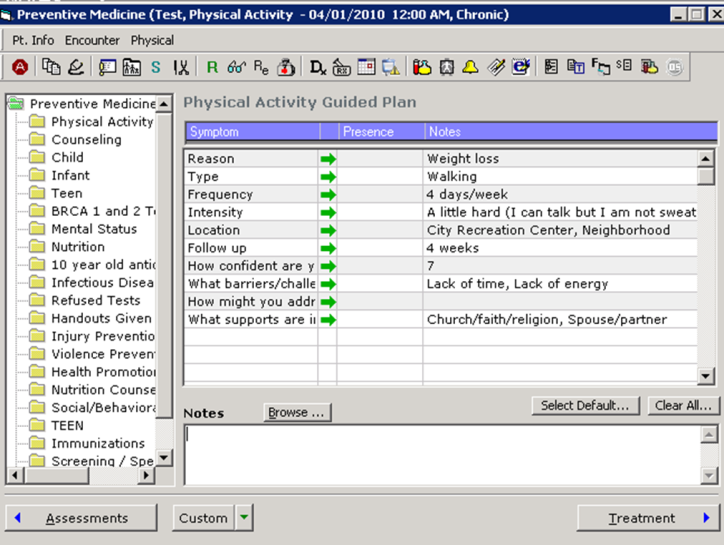
**

**Figure 4: Order Sets Page**

**
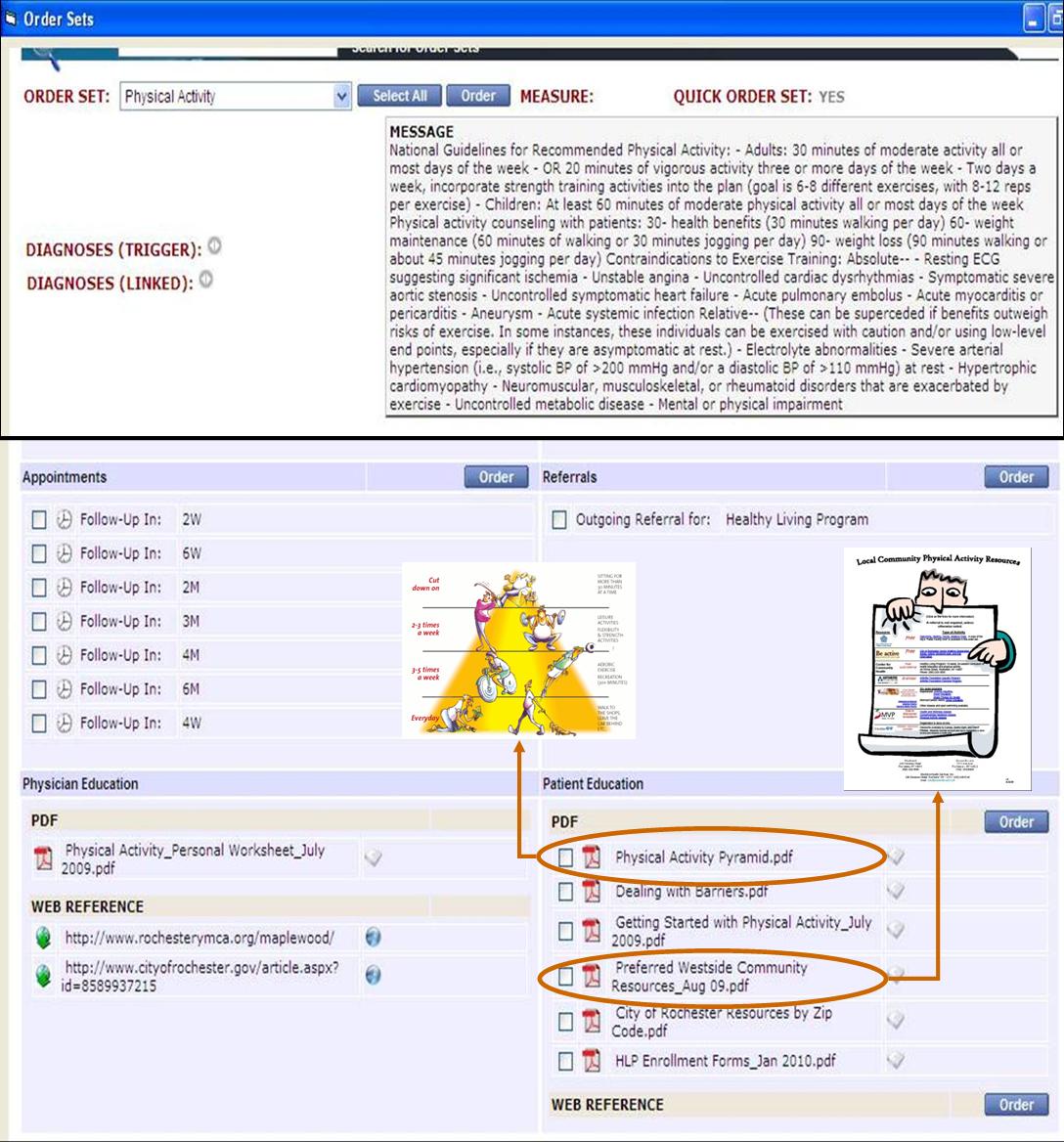
**

**Figure 5: Clinician Referrals Tab (link) to community program**

**
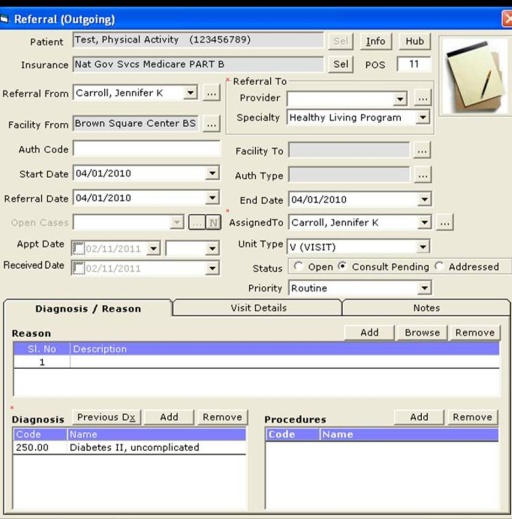
**
